# Supplementary material for: MYC induces CDK4/6 inhibitors resistance by promoting pRB1 degradation
Source: Nat Commun. 2024 Feb 29;15:1871. doi: 10.1038/s41467-024-45796-w (PMC10904810; doi:10.1038/s41467-024-45796-w)
Supplement: Supplementary file 1 — Supplementary Information [file 41467_2024_45796_MOESM1_ESM.pdf]

Supplementary Information

MYC induces CDK4/6 inhibitors resistance by promoting RB1 degradation

Jian Ma, Lei Li, Bohan Ma, Tianjie Liu, Zixi Wang, Qi Ye, Yunhua Peng, Bin Wang, Yule Chen, Shan Xu, Ke Wang, Fabin Dang, Xinyang Wang, Zixuan Zeng, Yanlin Jian, Zhihua Ren, Yizeng Fan, Xudong Li, Jing Liu, Yang Gao, Wenyi Wei and Lei Li

This PDF file includes:

Supplemental Figure 1 to 15

Supplementary Fig. 1

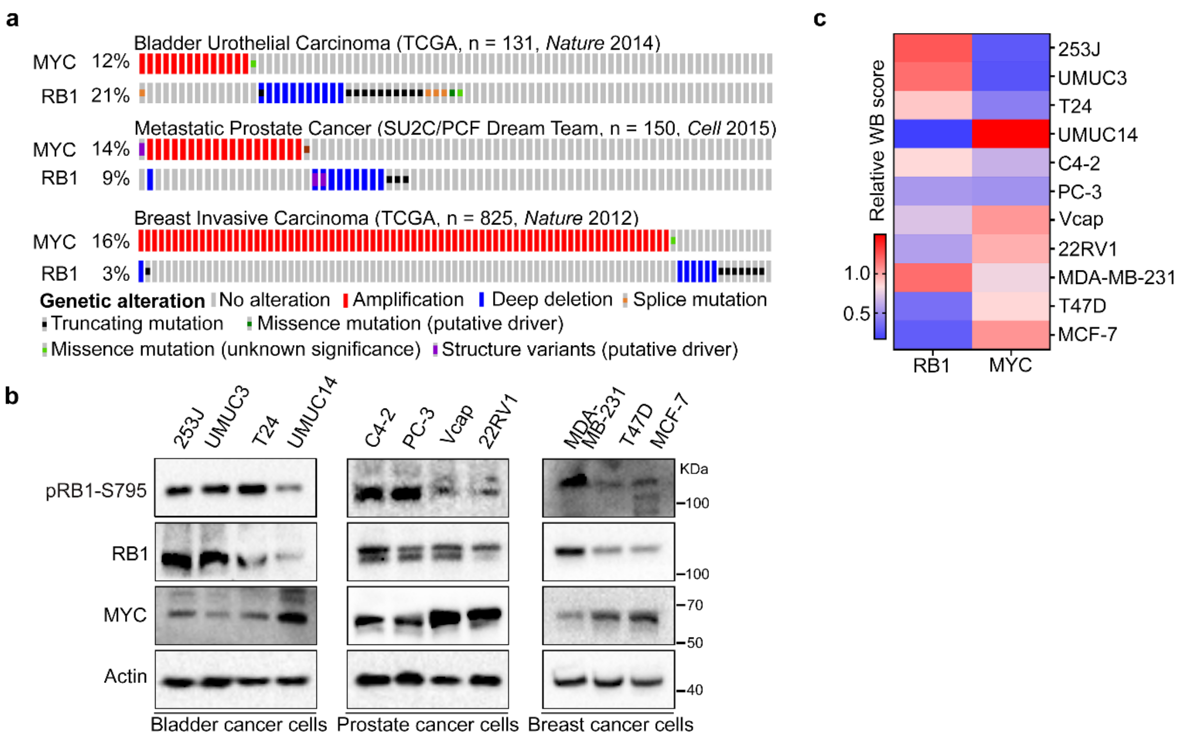

Supplementary Fig. 1 The correlation between MYC and RB1. Related to Fig. 1. a Genetic alteration of MYC and RB1 in the indicated cohorts from The Cancer Genome Atlas (TCGA) datasets. **b** Western blot analysis of MYC and RB1 from indicated cell lines. **c** Heatmap of quantification of protein intensity of MYC and RB1 in **b.** Source data are provided in this paper. Similar results for (**b**) panels were obtained in three independent experiments.

Supplementary Fig. 2

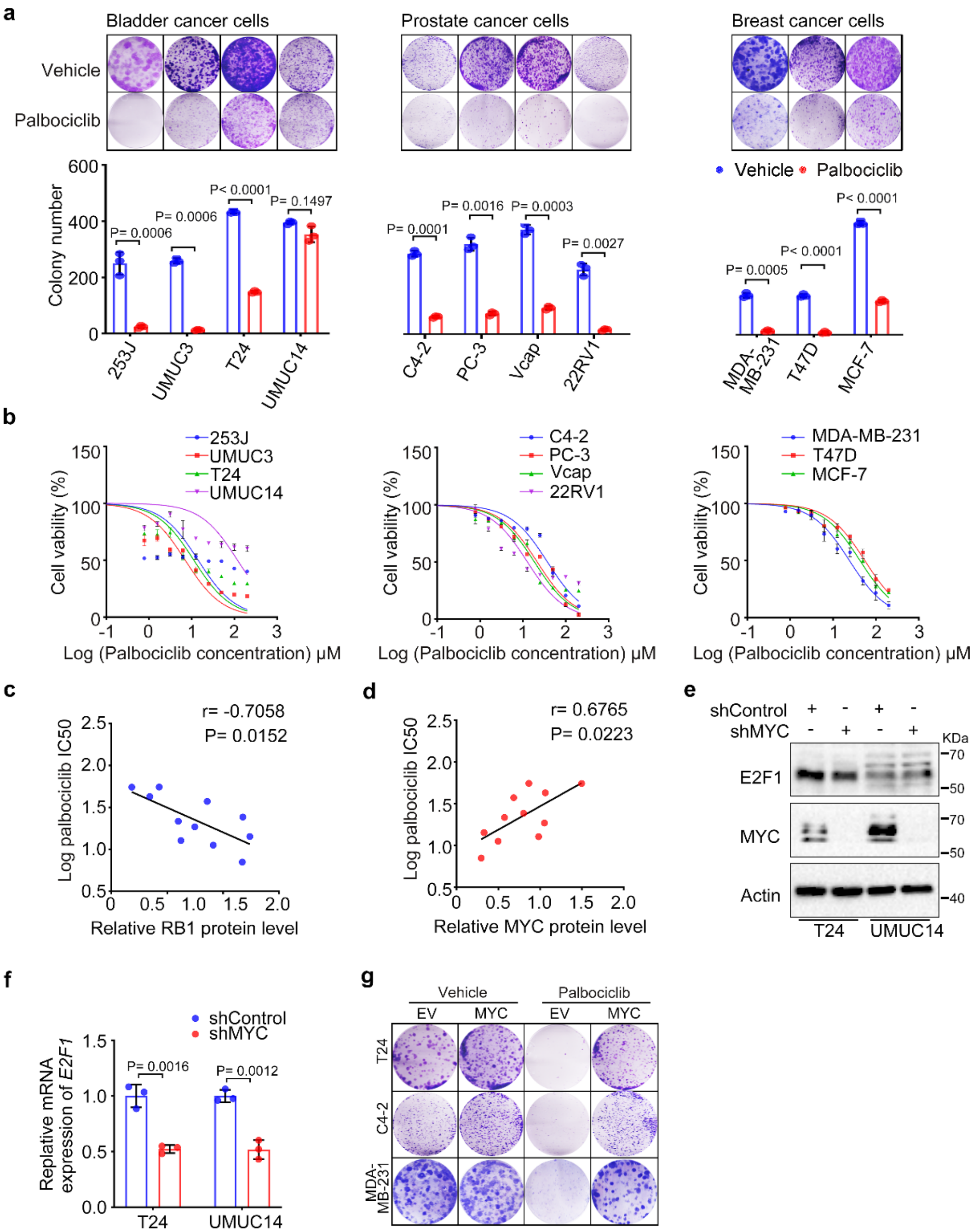

Supplementary Fig. 2 High MYC expression drives the acquired resistance of CDK4/6i. Related to Fig. 1. a Bladder, prostate, breast cancer cell lines treated with vehicle or palbociclib were harvested for colony formation assay(top). Data were shown as the mean ± SD of three independent experiments (n = 3) (bottom). Two-tailed unpaired Student's t-test. P values based on the order of appearance: 0.0006, 0.0006, 2.61E-06 and 0.1497; 0.0001, 0.0016, 0.0003 and 0.0027; 0.0005, 3.56E-05 and 8.3E-06. **b** Indicated cell lines treated with vehicle or palbociclib

were harvested for cell viability assay. Data were shown as the mean ± SEM of three independent experiments (n = 3). **c, d** Correlation analysis of RB1(**c**) and MYC (**d**) protein levels and IC50 of palbociclib in bladder, prostate, breast cancer cell lines. **e, f** Control or MYC knockdown T24 and UMUC14 cells were harvested for western blotting (**e**), and RT- qPCR (**f**). In **f**, data were shown as the mean ± SD of three independent experiments (n = 3). Two-tailed unpaired Student's t-test. P values based on the order of appearance: 0.0016, and 0.0012. **g** Control or MYC overexpressed T24, PC-3, MDA-MB-231 cells were harvested for colony formation assay. Source data are provided in this paper. Similar results for (**e**) panels were obtained in three independent experiments.

Supplementary Fig. 3

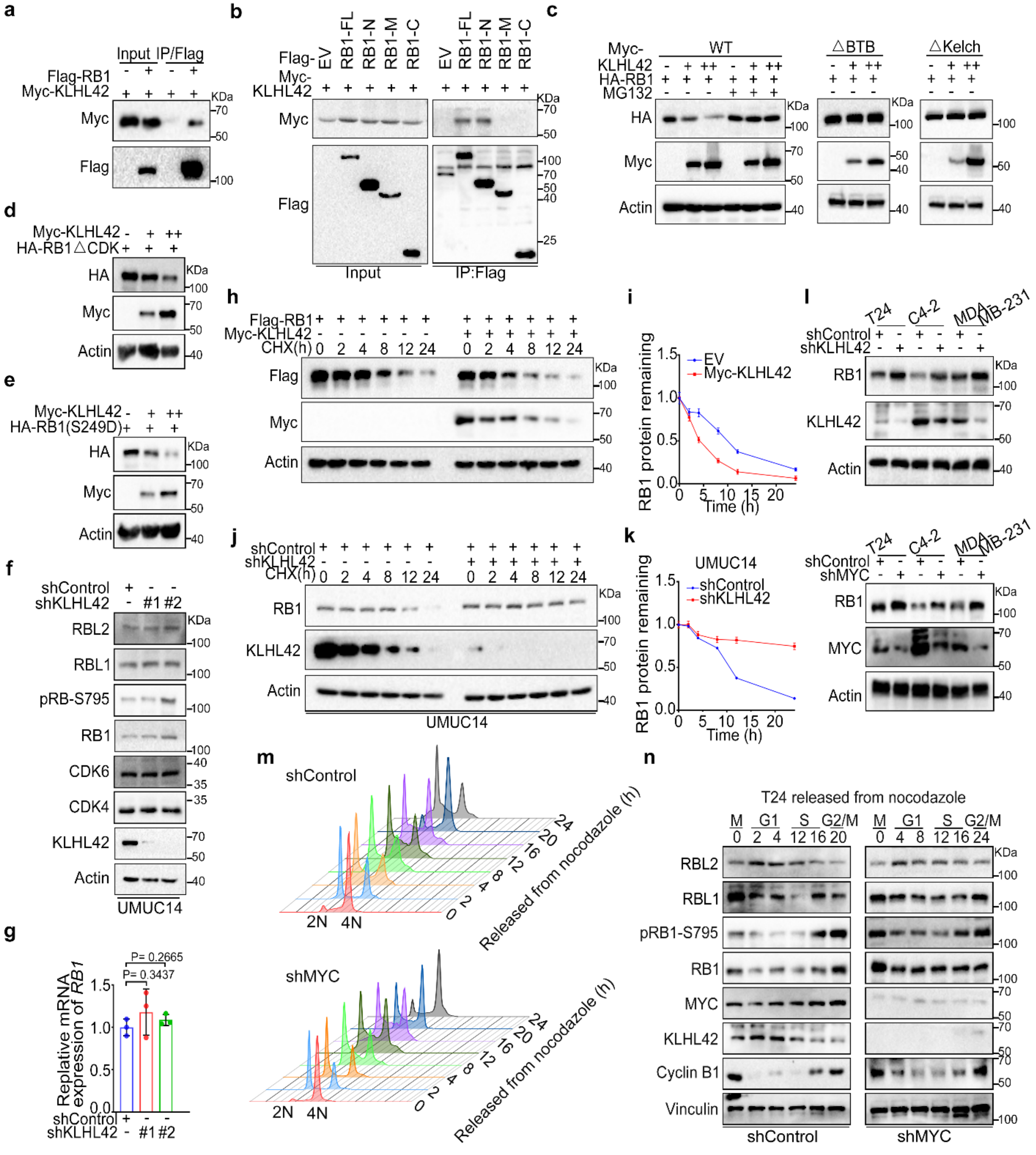

Supplementary Fig. 3 The E3 ubiquitin ligase KLHL42 interacts with RB1 and induces RB1 degradation in MYC-expressing cells. Related to Fig. 3. a T24 cells transfected with indicated plasmids were harvested for immunoprecipitation (IP) under denaturing condition and immunoblotting. **b** 293T cells transfected with the indicated plasmids were harvested for immunoprecipitation under denaturing conditions and subjected to immunoblotting. **c** 293T cells were transfected with indicated plasmids were harvested for western blotting. **d, e** 293T cells

were transfected with indicated plasmids were harvested for western blotting. **f**, **g** Control or KLHL42 knockdown UMUC14 cells were harvested for western blotting (**f**), and RT- qPCR (**g**). In **g**, data were shown as the mean ± SD of three independent experiments (n = 3). Two-tailed unpaired Student's t-test. P values based on the order of appearance: 0.3437, and 0.2665. **h, i** T24 cells were transfected with or without Myc-tagged KLHL42 for 24 h followed by treatment of 200 µg/µl CHX for western blot (**h**). Protein bands were quantified in **i**. In **i**, data were shown as the mean ± SD of three independent experiments (n = 3). **j, k** Control or KLHL42-knockdown UMUC14 cells were treated with 200 µg/µl CHX for western blot (**j**). Protein bands were quantified in **k**. In **k**, data were shown as the mean ± SD of three independent experiments (n = 3). **l** T24, C4-2, and MDA-MB-231 cells with KLHL42 or MYC knockdown were harvested for western blotting with the indicated antibodies. **m, n** T24 cells were synchronized in M phase by nocodazole. Cells were released and harvested at the indicated time points for FACS-based cell cycle analysis (**m**) and WB (**n**). Source data are provided in this paper. Similar results for (**a, b, c, d, e, f, h, j, l** and **n**) panels were obtained in three independent experiments.

Supplementary Fig. 4

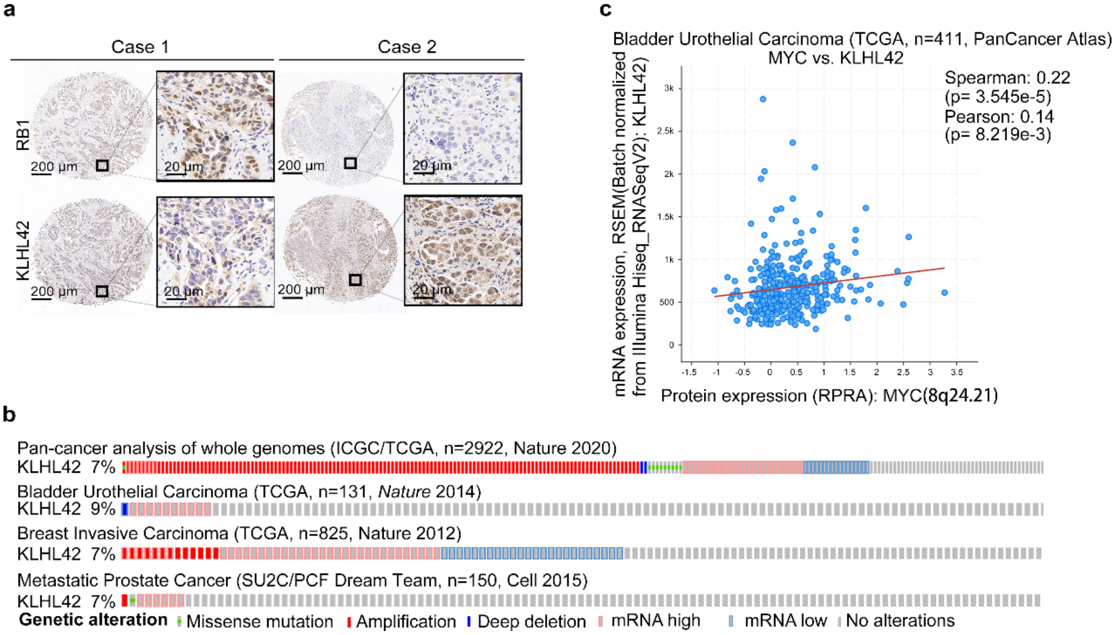

Supplementary Fig. 4 The correlation between KLHL42, MYC and RB1 in bladder cancer tissues and TCGA data sets. Related to Fig. 3 a Representative images of IHC analysis with anti-KLHL42 and anti-RB1 antibodies on TMA (n = 40 TMA elements) tissue sections. Scale bar in 10 X fields: 200 µm; Scale bar in 40 X fields: 20 µm. **b** Genetic alterations and mRNA expression of KLHL42 in the indicated cohorts from TCGA datasets. **c** Correlation analysis of protein expression of MYC and mRNA expression of KLHL42 from TCGA datasets. Source data are provided in this paper. Source data are provided in this paper.

Supplementary Fig. 5

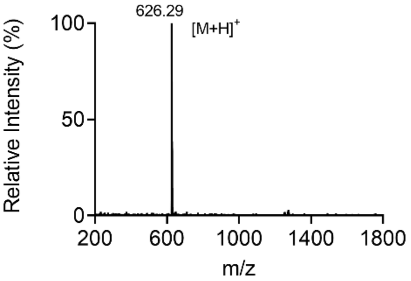

Supplementary Fig. 5 The high-resolution mass spectrometry of A80.2HCl. Related to Fig. 5

Supplementary Fig. 6

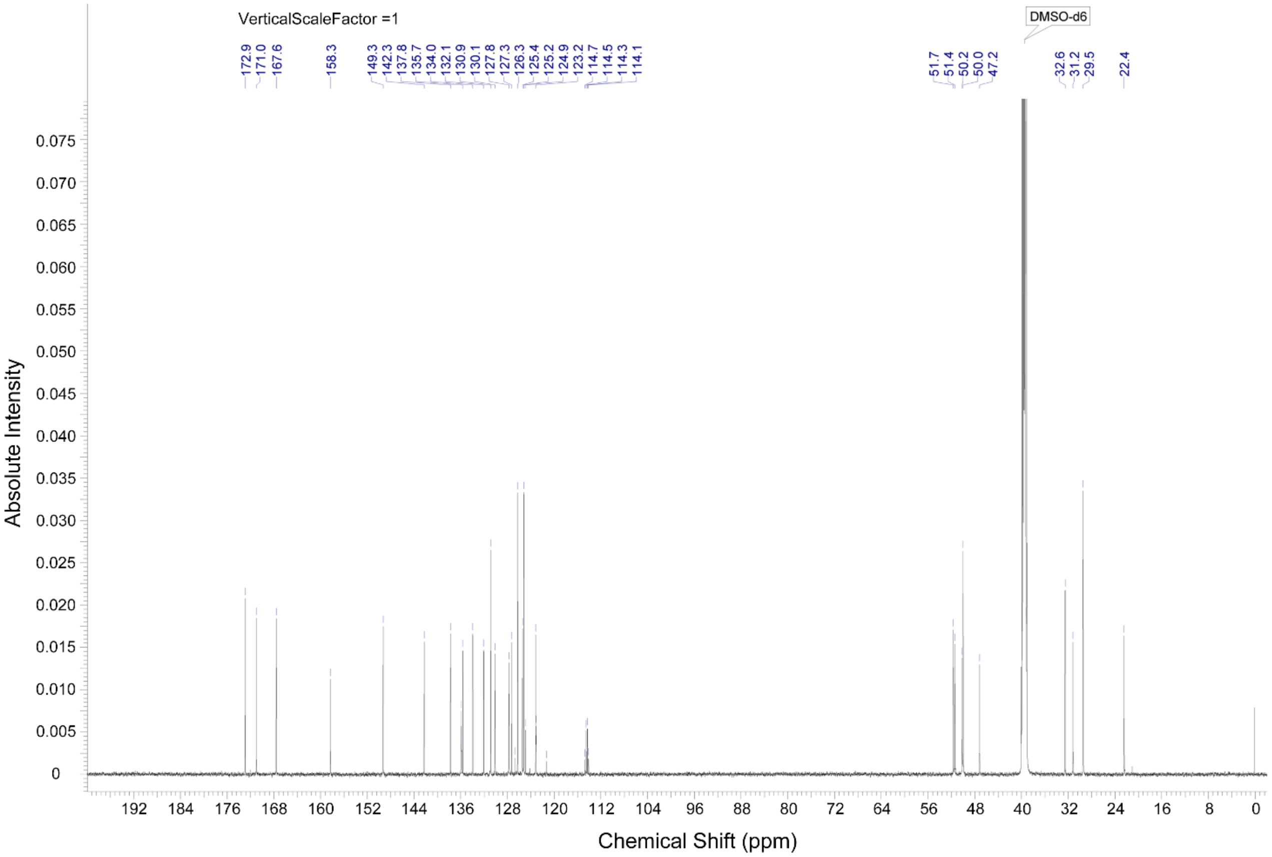

Supplementary Fig. 6 The 13C-NMR analysis of A80.2HCl. Related to Fig. 5

^{13}C NMR (151 MHz, *DMSO-d*$_6$) δ ppm 22.4 (1 C), 29.5 (2 C), 31.2 (1 C), 32.6 (1 C), 47.2 (1 C), 50.0 (2 C), 50.2 (1 C), 51.4 (1 C), 51.7 (1 C), 114.12, 114.32, 114.53, 114.73 (1 C), 123.2 (1 C), 121.32, 123.13, 124.93, 126.74 (1 C), 125.2 (1 C), 125.4 (1 C), 126.3 (1 C), 127.3 (1 C), 127.8 (1 C), 130.1 (1 C), 130.9 (1 C), 132.1 (1 C), 134.0 (1 C), 135.7 (1 C), 135.86, 135.89, 135.92, 135.95 (1 C), 137.8 (1 C), 142.3 (1 C), 149.3 (1 C), 158.3 (1 C), 167.6 (1 C), 171.0 (1 C), 172.9 (1 C)

Supplementary Fig. 7

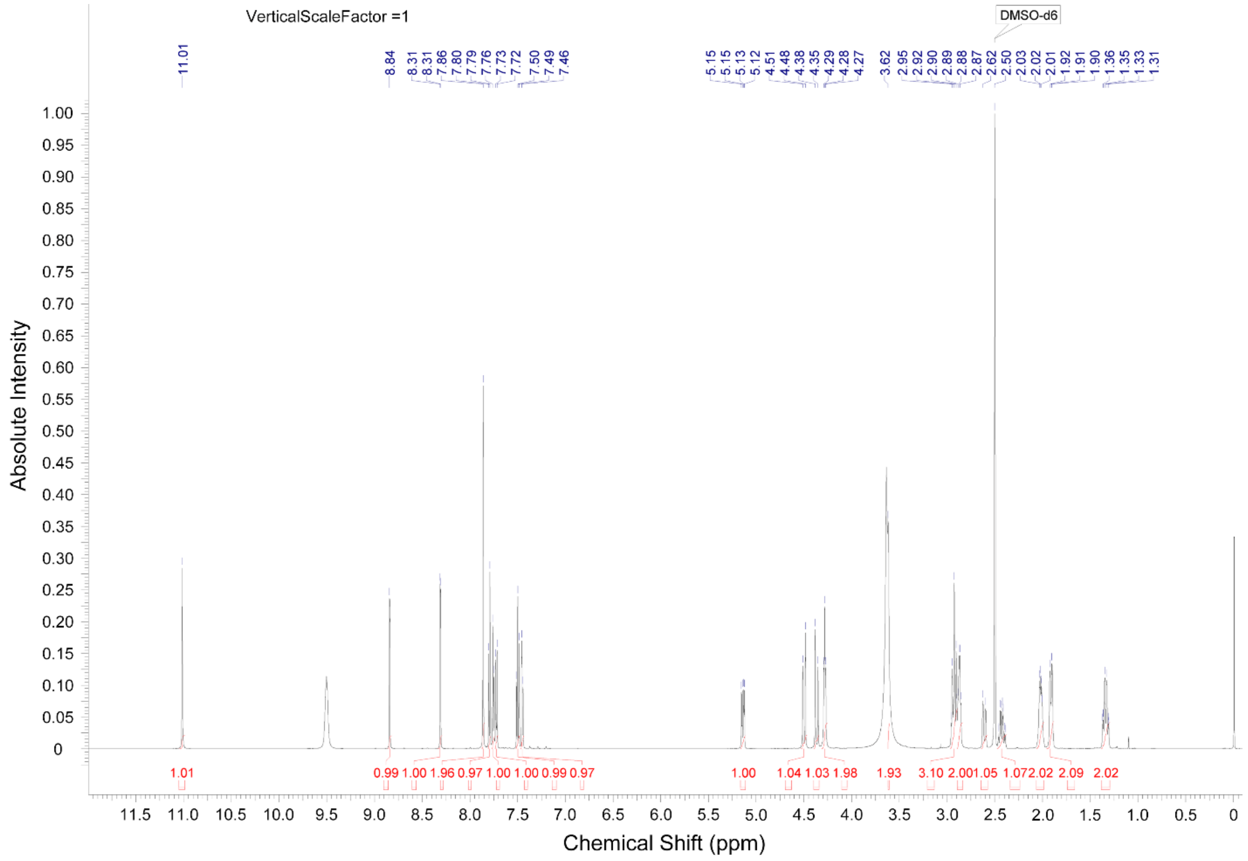

Supplementary Fig. 7 The 1H-NMR analysis of A80.2HCl. Related to Fig. 5

^{1}H NMR (600 MHz, *DMSO-d_6*) δ ppm 1.30 - 1.40 (m, 2 H), 1.89 - 1.95 (m, 2 H), 1.99 - 2.07 (m, 2 H), 2.44 (qd, *J*=13.24, 4.31 Hz, 1 H), 2.62 (d, *J*=16.87 Hz, 1 H), 2.85 - 2.90 (m, 2 H), 2.90 - 2.97 (m, 3 H), 3.63 (d, *J*=12.65 Hz, 2 H), 4.29 (t, *J*=5.23 Hz, 2 H), 4.37 (d, *J*=17.42 Hz, 1 H), 4.51 (d, *J*=17.42 Hz, 1 H), 5.15 (dd, *J*=13.30, 5.04 Hz, 1 H), 7.44 - 7.48 (m, 1 H), 7.49 - 7.53 (m, 1 H), 7.73 (d, *J*=7.89 Hz, 1 H), 7.76 (d, *J*=7.89 Hz, 1 H), 7.80 (d, *J*=7.89 Hz, 1 H), 7.87 (s, 2 H), 8.32 (d, *J*=2.20 Hz, 1 H), 8.85 (d, *J*=2.02 Hz, 1 H), 11.02 (s, 1 H).

Supplementary Fig. 8

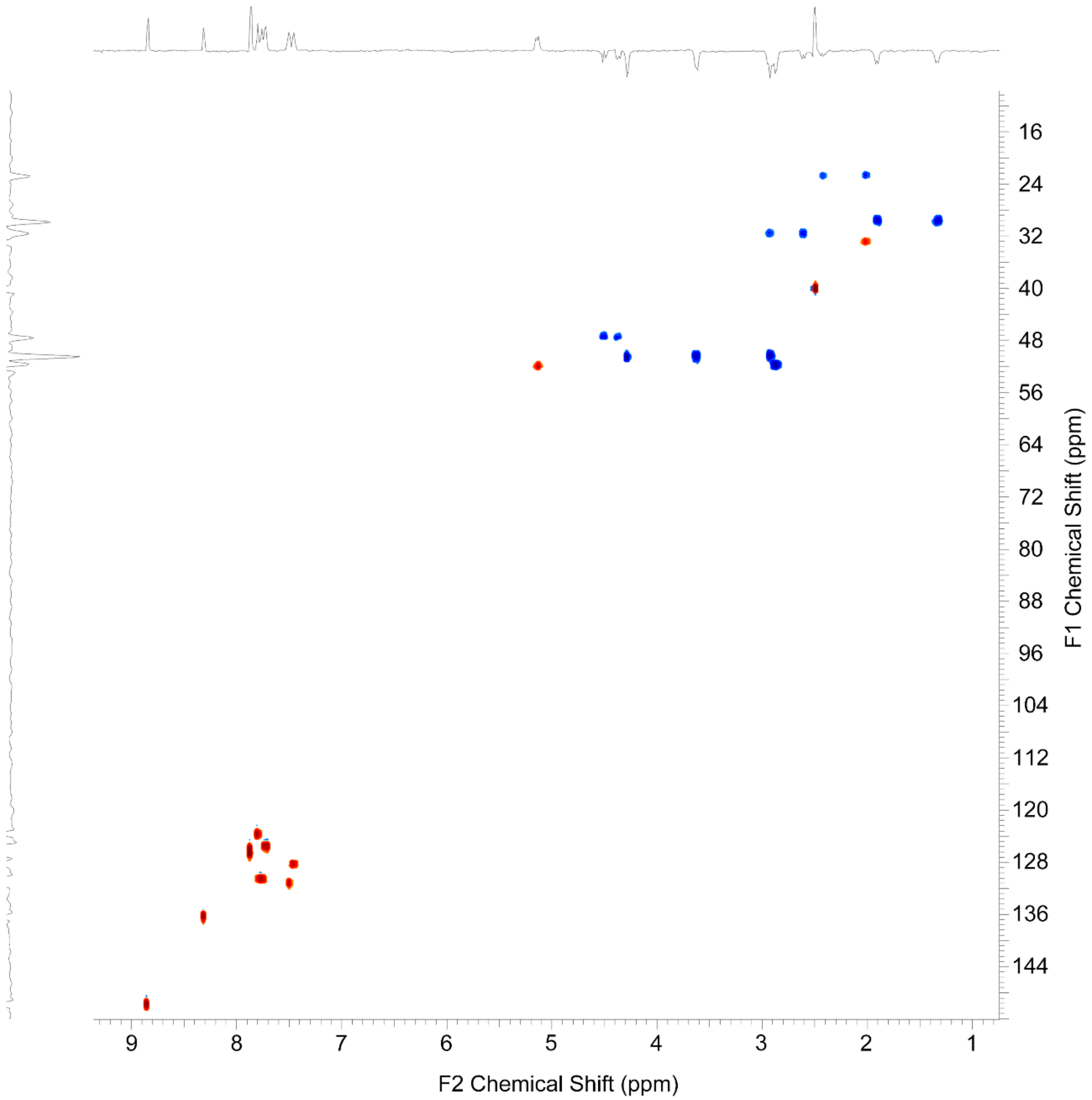

Supplementary Fig. 8 The two-dimensional NMR spectroscopy analysis of A80.2HCl. Related to Fig. 5

Supplementary Fig. 9

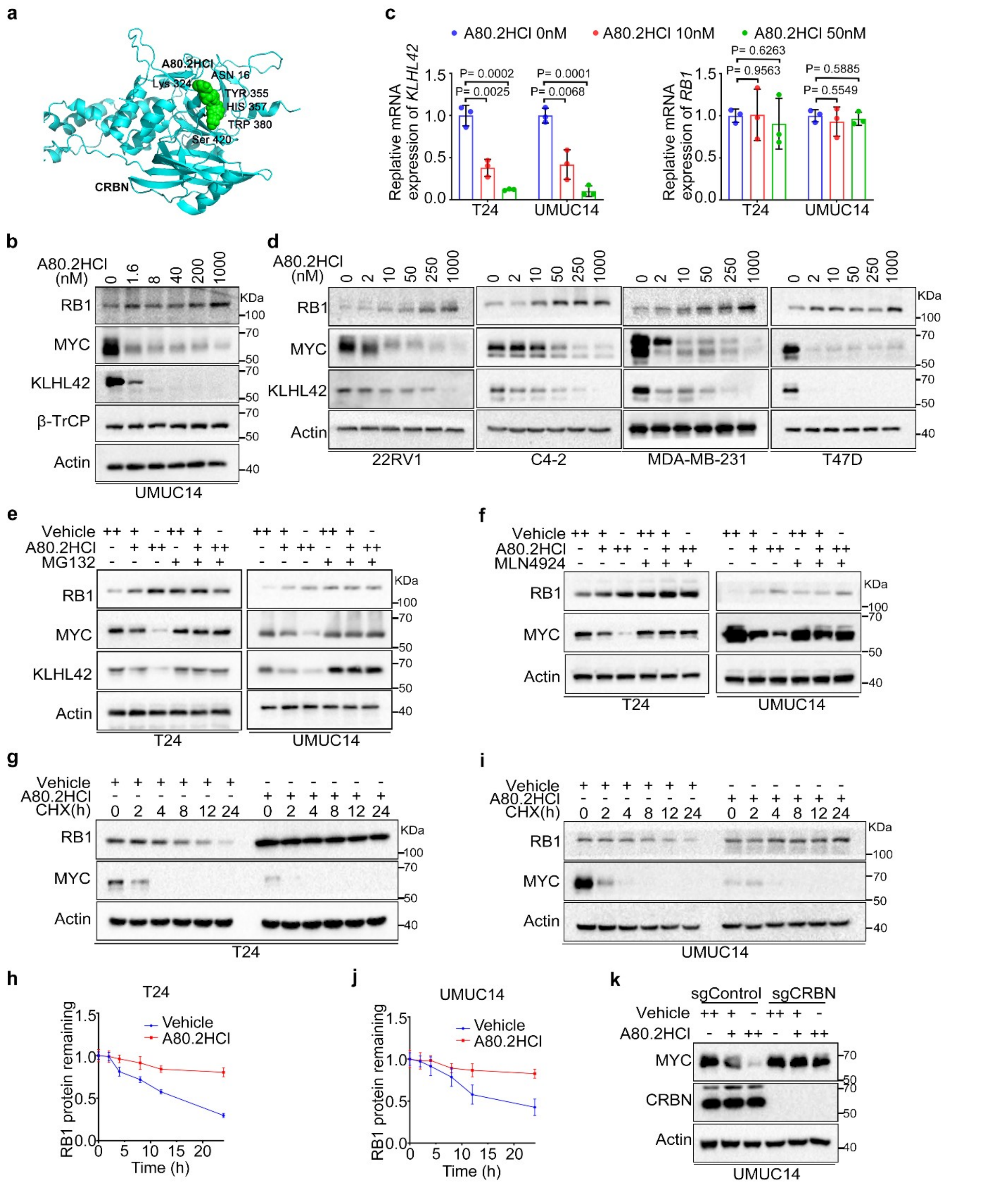

Supplementary Fig. 9 Identification of A80.2HCl as a novel molecule degrader of MYC. Related to Fig. 5. a Docking simulation cartoon illustrating that A80.2HCl (green) specifically bound to CRBN (blue), the key residues at the binding interface were provided. **b** UMUC14 cells

treated with increased quantities of A80.2HCl were harvested for western blotting. **c** T24 and UMUC14 cells treated with increased quantities of A80.2HCl were harvested for RT- qPCR. Data were shown as the mean ± SD of three independent experiments (n = 3). Two-tailed unpaired Student's t-test. P values based on the order of appearance: 0.0025, 0.0002, 0.0068 and 0.0001; 0.9563, 0.6263, 0.5549 and 0.5885. **d** 22RV1, C4-2, MDA-MB-231 and T47D cells treated with increased quantities of A80.2HCl were harvested for western blotting. **e, f** T24 and UMUC14 cells treated with indicated drugs were harvested for western blotting. **g, h** T24 cells treated with vehicle or A80.2HCl for 24 h followed by treatment of 200 μg/μl CHX were harvested for western blot (**g**). Protein bands were quantified in **h**. In **h**, data were shown as the mean ± SD of three independent experiments (n = 3). **i, j** UMUC14 cells treated with vehicle or A80.2HCl for 24 h followed by treatment of 200 μg/μl CHX were harvested for western blot (**i**). Protein bands were quantified in **j**. In **j**, data were shown as the mean ± SD of three independent experiments (n = 3). **k** $CRBN^{WT}$ and $CRBN^{KO}$ UMUC14 cells treated with vehicle and A80.2HCl were harvested for western blotting. Source data are provided in this paper. Similar results for (**b, c, d, e, f, g, i** and **k**) panels were obtained in three independent experiments.

Supplementary Fig. 10

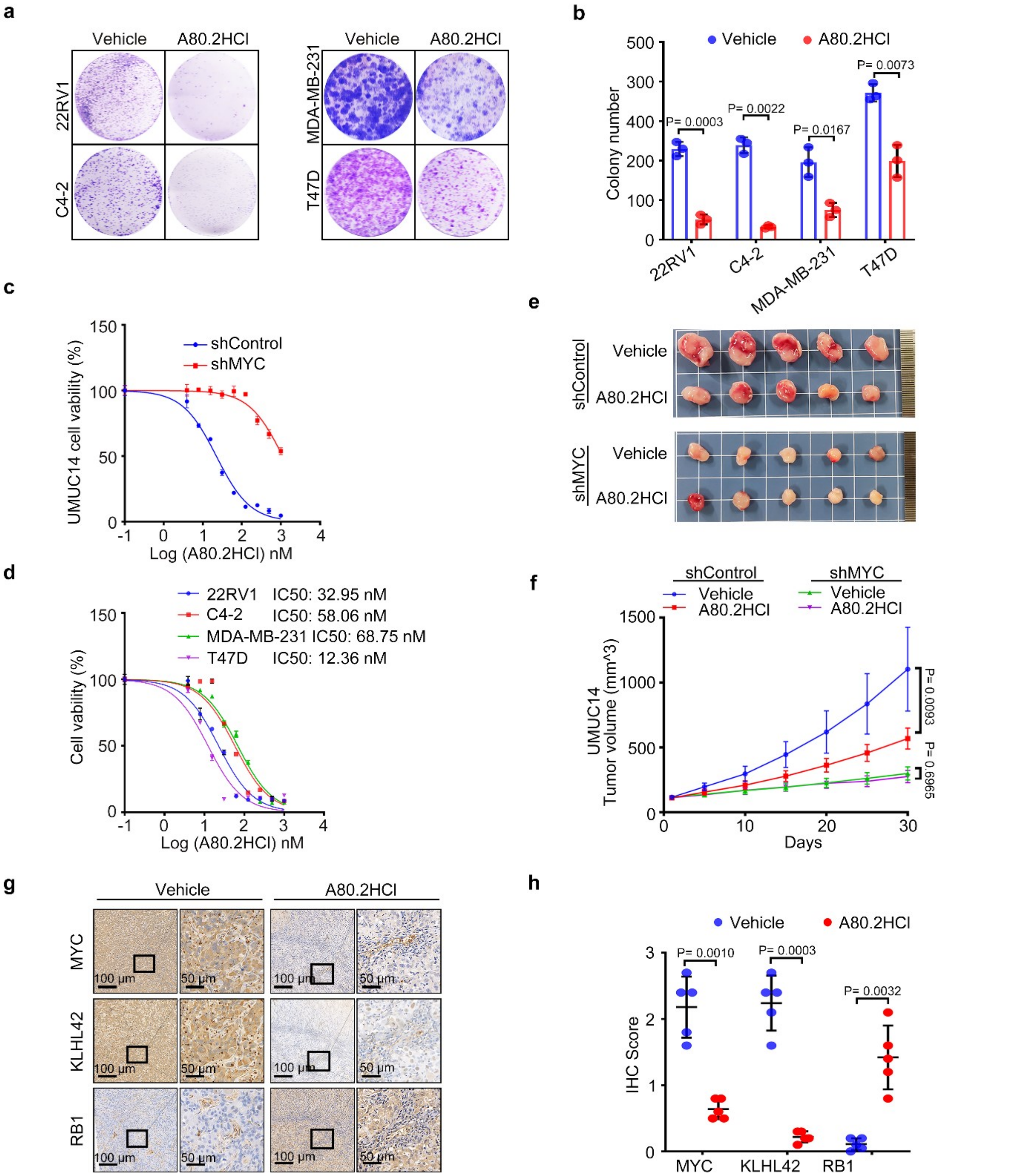

Supplementary Fig. 10 Identification of A80.2HCl as a novel molecule degrader of MYC. Related to Fig. 5. a, b 22RV1, C4-2, MDA-MB-231 and T47D cells treated with vehicle or A80.2HCl were harvested for colony formation assay (**a**). In **b**, data were shown as the mean ± SD of three independent experiments (n = 3). Two-tailed unpaired Student's t-test. P values based on the order of appearance: 0.0003, 0.0022, 0.0167 and 0.0073. **c** Control or MYC

knockdown UMUC14 cells treated with A80.2HCl were harvested for cell viability assay. Data were shown as the mean ± SEM of three independent experiments (n = 3). **d** 22RV1, C4-2, MDA-MB-231 and T47D cells treated with vehicle or A80.2HCl were harvested for cell viability assay. Data were shown as the mean ± SEM of three independent experiments (n = 3). **e, f** Control or MYC knockdown UMUC14 cells were injected s.c. into the right flank of NSG mice and treated with the indicated drugs. Tumor volume was measured at indicated time points. Tumors were harvested and photographed at day 30 (**e**). In **f**, data were shown as the mean ± SD of five mice (n = 5). Two-way ANOVA (two-sided). P values based on the order of appearance: 0.0093 and 0.6965. **g, h** Representative images of IHC analysis with anti-RB1, anti-MYC and anti-KLHL42 antibodies on T24 xenograft tumors from Fig. 5m (**g**) and the quantification of IHC data are shown in **h**. In **h**, data were shown as the mean ± SD of three independent experiments (n = 3). Two-tailed unpaired Student's t-test. P values based on the order of appearance: 0.0010, 0.0003 and 0.0032. Source data are provided in this paper.

Supplementary Fig. 11

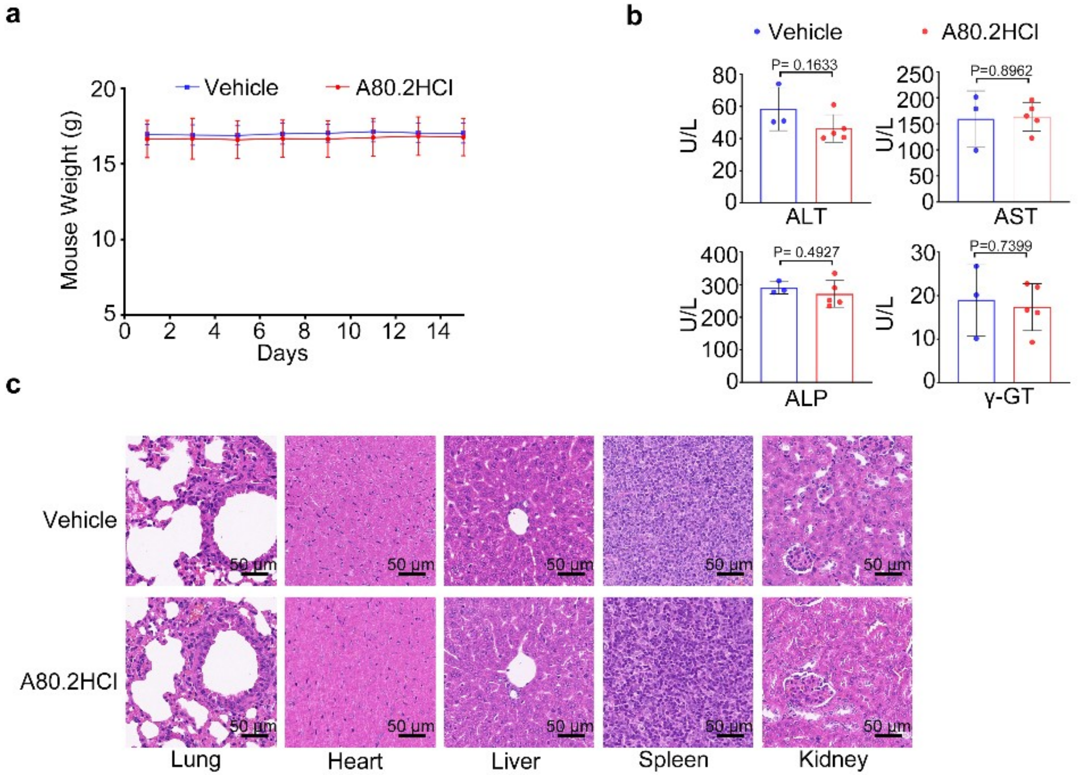

Supplementary Fig. 11 Safety evaluation of A80.2HCl in vivo. Related to Fig. 5. a Mouse body weights were detected after 15-day treatment of A80.2HCl. Data were shown as the mean ± SD (Vehicle n =5, A80.2HCl n =5). **b** Biochemical analysis of serum after 15-day treatment of A80.2HCl. Data were shown as the mean ± SD of independent experiments (Vehicle n =3, A80.2HCl n =5). Two-tailed unpaired Student's t-test. P values based on the order of appearance: 0.1633, 0.8962, 0.4927 and 0.7399. **c** Representative H&E staining photograph of lung, heart, liver, spleen, and kidney sections from mice after the 15-day treatment of A80.2HCl. Scale bar, 50 µm. Source data are provided in this paper.

Supplementary Fig. 12

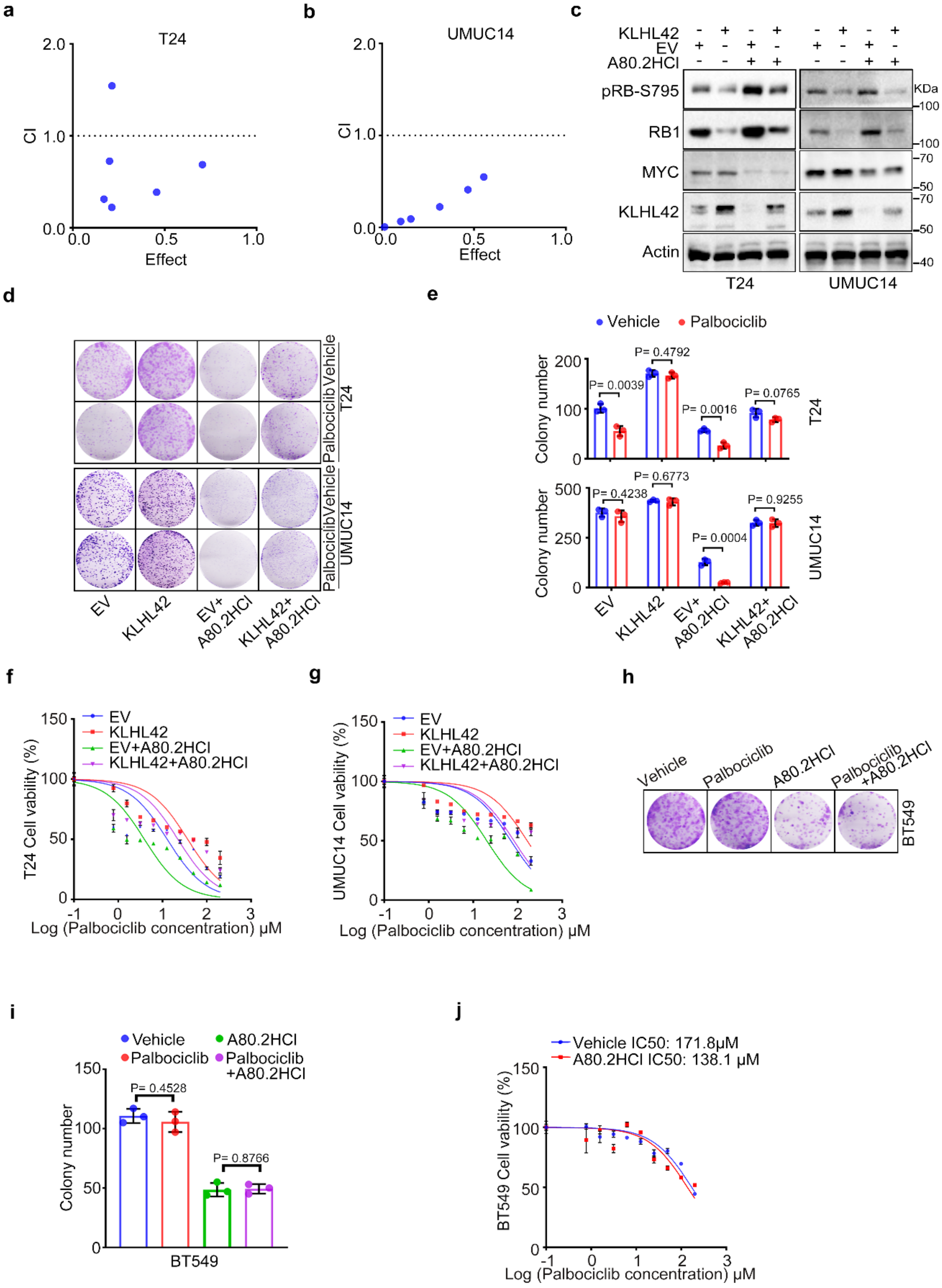

Supplementary Fig. 12 A80.2HCl potentiates the therapeutic efficacy of CDK4/6 inhibitors. Related to Fig. 6. a, b Combination Index plot of palbociclib and A80.2HCl treatment of T24(**a**) andUMUC14(**b**) cells. T24 and UMUC14 cells were co-treated with 10nM A80.2HCl and diferent concentrations of palbocilib for 24h, and the viability of the cells was measured by the MTT assay. The dose-effect profiles were used to calculate Combination Index (C.I.) values as described in the Materials and Methods section. The data points below or above the line indicate synergistic or antagonistic drug interactions, respectively. **c** Control or KLHL42-overexpressing T24 and UMUC14 cells treated with vehicle or A80.2HCl were harvested for western blotting. **d, e** Control or KLHL42-overexpressing T24 and UMUC14 cells treated with vehicle or A80.2HCl were harvested for colony formation assay (**d**). In **e**, data were shown as the mean ± SD of three independent experiments (n = 3). Two-tailed unpaired Student's t-test. P values based on the order of appearance: 0.0039, 0.4792, 0.0016 and 0.0765; 0.4238, 0.6773, 0.0004 and 0.9255. **f, g** Control or KLHL42-overexpressing T24 (**f**) and UMUC14 (**g**) cells treated with vehicle or A80.2HCl were harvested for the cell viability assay. Data were shown as the mean ± SEM of three independent experiments (n = 3) **h, i** RB-deficient cells BT549 treated with the indicated drugs were harvested for colony formation assay (**h**). In **i**, data were shown as the mean ± SD of three independent experiments (n = 3). Two-tailed unpaired Student's t-test. P values based on the order of appearance: 0.4528 and 0.8766. **j** RB-deficient cells BT549 treated with the indicated drugs were harvested for the cell viability assay. Data were shown as the mean ± SEM of three independent experiments (n = 3). Source data are provided in this paper. Similar results for (**c**) panels were obtained in three independent experiments.

Supplementary Fig. 13

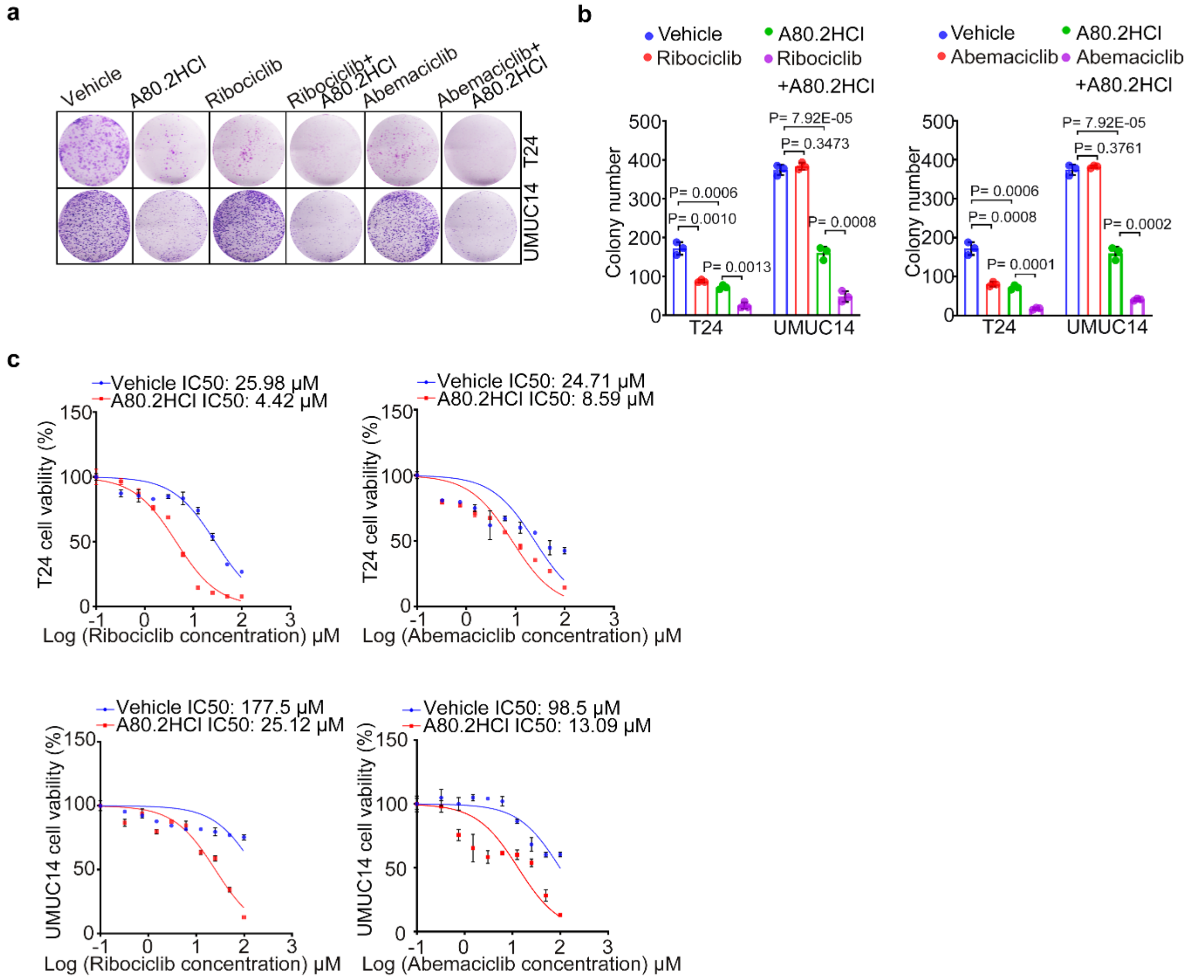

Supplementary Fig. 13 A80.2HCl potentiates the therapeutic efficacy of CDK4/6 inhibitors. Related to Fig. 6. a, b T24 and UMUC14 cells treated with the indicated drugs were harvested for colony formation assay (**a**). In **b**, data were shown as the mean ± SD of three independent experiments (n = 3). Two-tailed unpaired Student's t-test. P values based on the order of appearance: 0.0010, 0.0006, 0.0013, 0.3473,7.92E-05 and 0.0008; 0.0008, 0.0006, 0.0001, 0.3761, 7.92E-05 and 0.0002. **c** T24 (top) and UMUC14 (bottom) cells treated with the indicated drugs were harvested for cell viability assays. Data were shown as the mean ± SEM of three independent experiments (n = 3). Source data are provided in this paper.

Supplementary Fig. 14

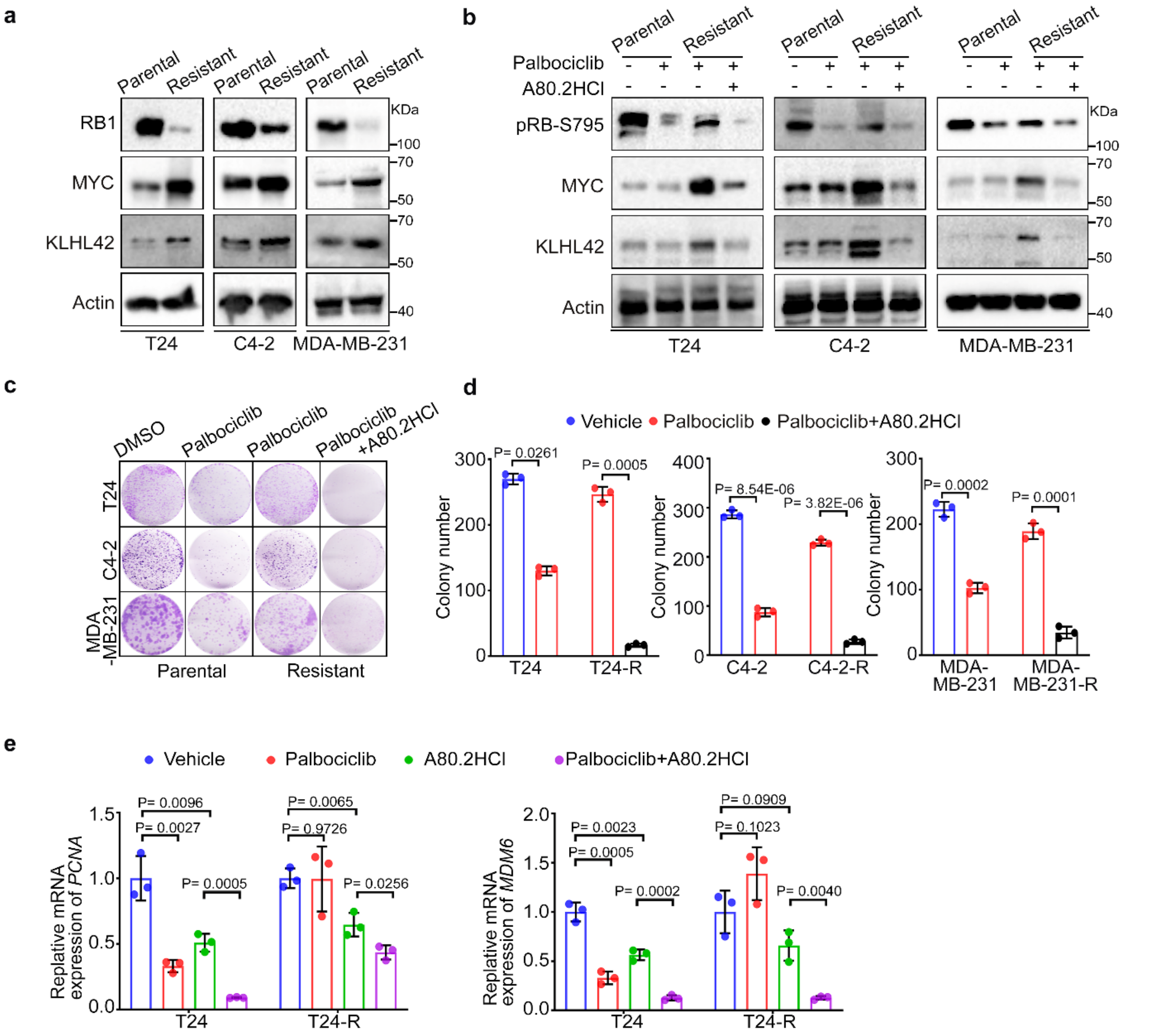

Supplementary Fig. 14 A80.2HCl re-sensitized the resistant cancer cells to CDK4/6i treatment. Related to Fig. 6. a Western blot analysis of expression of RB1, MYC and KLHL42 in normal parental and CDK4/6i-resistant daughter cancer cells. **b** Western blot analysis showing that addition of A80.2HCl potentiated the suppressing efficacy of palbociclib in CDK4/6i resistant cells. **c, d** Combination of A80.2HCl potentiated the efficacy of palbociclib in suppressing colony formation in CDK4/6i-resistant daughter cancer cells. In **d**, data were shown as the mean ± SD of three independent experiments (n = 3). Two-tailed unpaired Student's t-test. P values based on the order of appearance: 0.0261 and 0.0005; 8.54E-06 and 3.82E-06; 0.0002 and 0.0001. **e** Normal parental and CDK4/6i-resistant daughter cancer cells treated with the indicated drugs were harvested for RT- qPCR. Data were shown as the mean ± SD of three independent experiments (n = 3). Two-tailed unpaired Student's t-test. P values based on the order of appearance: 0.0027, 0.0096, 0.0005, 0.9726, 0.0065 and 0.0256; 0.0005, 0.0023, 0.0002,

0.1023, 0.0909 and 0.0040. Source data are provided in this paper. Similar results for (**a** and **b**) panels were obtained in three independent experiments.

Supplementary Fig. 15

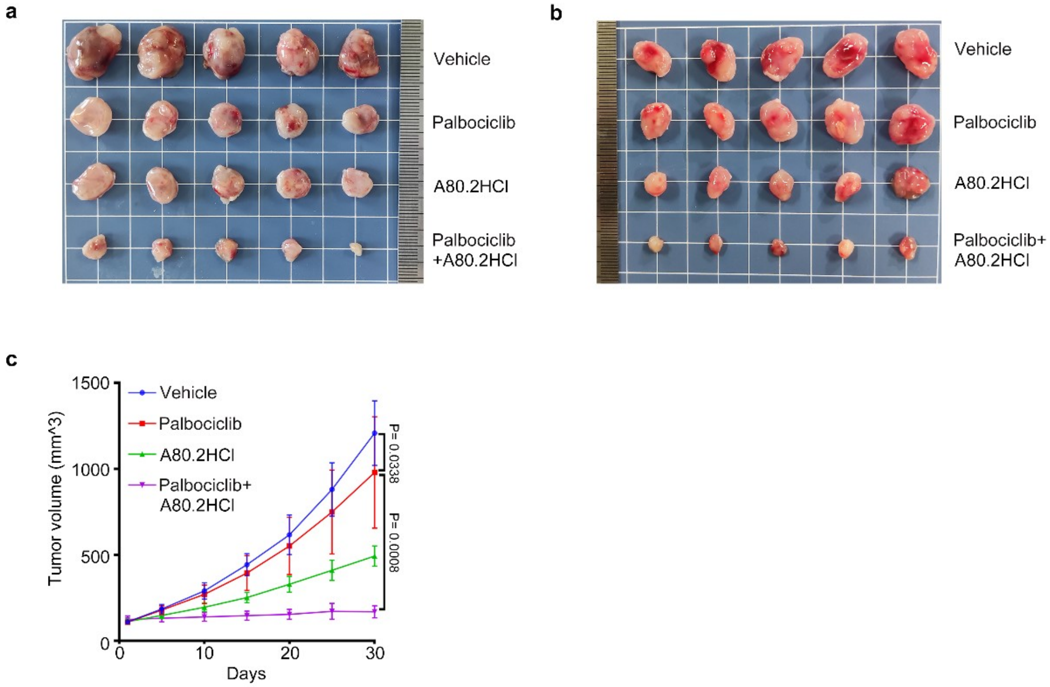

Supplementary Fig. 15 Effect of A80.2HCl, alone or in combination with CDK4/6i in tumor cells. Related to Fig. 6. a T24 cells were injected s.c. into the right flank of NSG mice and treated with the indicated drugs. Tumor volume was measured at indicated time points. Tumors were harvested and photographed at day 30. **b, c** UMUC14 cells were injected s.c. into the right flank of NSG mice. Tumor volume was measured at indicated time points. Tumors were harvested and photographed at day 30 (**b**). In **c**, data were shown as the mean ± SD of five mice (n = 5). Two-way ANOVA (two-sided). P values based on the order of appearance: 0.0338 and 0.0008. Source data are provided in this paper.
