# Supplementary material for: MYC induces CDK4/6 inhibitors resistance by promoting pRB1 degradation
Source: Nat Commun. 2024 Feb 29;15:1871. doi: 10.1038/s41467-024-45796-w (PMC10904810; doi:10.1038/s41467-024-45796-w)
Supplement: Supplementary file 2 — Reporting Summary [file 41467_2024_45796_MOESM2_ESM.pdf]

nature portfolio

Corresponding author(s): Lei Li

Last updated by author(s): Dec 25, 2023

Reporting Summary

Nature Portfolio wishes to improve the reproducibility of the work that we publish. This form provides structure for consistency and transparency in reporting. For further information on Nature Portfolio policies, see our Editorial Policies and the Editorial Policy Checklist.

Statistics

For all statistical analyses, confirm that the following items are present in the figure legend, table legend, main text, or Methods section.

n/a	Confirmed	
☐	☒	The exact sample size (*n*) for each experimental group/condition, given as a discrete number and unit of measurement
☐	☒	A statement on whether measurements were taken from distinct samples or whether the same sample was measured repeatedly
☐	☒	The statistical test(s) used AND whether they are one- or two-sided<br>*Only common tests should be described solely by name; describe more complex techniques in the Methods section.*
☐	☒	A description of all covariates tested
☐	☒	A description of any assumptions or corrections, such as tests of normality and adjustment for multiple comparisons
☐	☒	A full description of the statistical parameters including central tendency (e.g. means) or other basic estimates (e.g. regression coefficient) AND variation (e.g. standard deviation) or associated estimates of uncertainty (e.g. confidence intervals)
☐	☒	For null hypothesis testing, the test statistic (e.g. *F*, *t*, *r*) with confidence intervals, effect sizes, degrees of freedom and *P* value noted<br>*Give P values as exact values whenever suitable.*
☒	☐	For Bayesian analysis, information on the choice of priors and Markov chain Monte Carlo settings
☐	☒	For hierarchical and complex designs, identification of the appropriate level for tests and full reporting of outcomes
☒	☐	Estimates of effect sizes (e.g. Cohen's *d*, Pearson's *r*), indicating how they were calculated

Our web collection on statistics for biologists contains articles on many of the points above.

Software and code

Policy information about availability of computer code

Data collection	CFX96 Touch™ System was used to perform quantitative PCR. Leica SCN400 was used to take IHC pictures. Microsoft Excel version 2010 was used to process the data.
Data analysis	Graphing and statistical analysis: GraphPad Prism 8.0 (GraphPad, Inc.) or Microsoft Office Excel 2010. Mass spectrometry analysis were processed with the UniProt human protein database (75,004 entries, download on 07-01-2020) using Protein Discoverer (Version 2.4.1.15, Thermo Fisher Scientific) and Mascot (Version 2.7.0, Matrix Science).The differential expression analysis of RNA-seq was performed using the DESeq2 (v1.30.1) Bioconductor package.

For manuscripts utilizing custom algorithms or software that are central to the research but not yet described in published literature, software must be made available to editors and reviewers. We strongly encourage code deposition in a community repository (e.g. GitHub). See the Nature Portfolio guidelines for submitting code & software for further information.

Data

Policy information about availability of data

All manuscripts must include a data availability statement. This statement should provide the following information, where applicable:

- Accession codes, unique identifiers, or web links for publicly available datasets
- A description of any restrictions on data availability
- For clinical datasets or third party data, please ensure that the statement adheres to our policy

All data associated with this study are present in the paper or the Supplementary Materials. The mass spectrometry proteomics raw data have been deposited to

the ProteomeXchange Consortium via the iProX partner repository with the dataset identifier PXD037479 (https://proteomecentral.proteomexchange.org/cgi/GetDataset?ID=PXD037479). The published MYC structure used in this study can be found in the Protein Data Bank under accession codes: 1A93. Raw sequencing data have been deposited in the National Center for Biotechnology Information Sequence Read Archive under the BioSample accession SAMN31422159 (https://www.ncbi.nlm.nih.gov/biosample/SAMN31422159) and BioProject accession PRJNA893398 (https://www.ncbi.nlm.nih.gov/bioproject/?term=PRJNA893398). Further information and requests for resources and reagents should be directed to and will be fulfilled by the lead contact, Lei Li (lilydr@163.com).

Research involving human participants, their data, or biological material

Policy information about studies with human participants or human data. See also policy information about sex, gender (identity/presentation), and sexual orientation and race, ethnicity and racism.

Reporting on sex and gender	The Bladder cancer tissue microarray slides were generated from both male and female patients without any gender bias. The tumor cells derived from breast cancer tissues (female-specific cancer type) for mini patient-derived xenograft were generated from only female patients, because this is a MYC amplification model.
Reporting on race, ethnicity, or other socially relevant groupings	The Bladder cancer tissue microarray slides bought from Shanghai Outdo Biotech Company; The mini PDX model were provided by Shanghai LIDE Biotech. All the experiments were designed without any biases on race, ethnicity, or other socially relevant groupings.
Population characteristics	The Bladder cancer tissue microarray slides bought from Shanghai Outdo Biotech Company. The slides were further confirmed through review of pathology reports and clinical records by trained personnel from the First Affiliated Hospital of Xi'an Jiaotong University. The mini PDX model were provided by Shanghai LIDE Biotech.
Recruitment	The Bladder cancer tissue microarray slides bought from Shanghai Outdo Biotech Company; The mini PDX model were provided from Shanghai LIDE Biotech.
Ethics oversight	The study protocol of clinical samples for bladder cancer tissue microarray slides was approved by the Institutional Ethics Committee of Shanghai Outdo Biotech Company; The mini PDX model protocol was approved by the Institutional Ethics Committee of Shanghai LIDE Biotech.

Note that full information on the approval of the study protocol must also be provided in the manuscript.

Field-specific reporting

Please select the one below that is the best fit for your research. If you are not sure, read the appropriate sections before making your selection.

- [x] Life sciences
- [ ] Behavioural & social sciences
- [ ] Ecological, evolutionary & environmental sciences

For a reference copy of the document with all sections, see nature.com/documents/nr-reporting-summary-flat.pdf

Life sciences study design

All studies must disclose on these points even when the disclosure is negative.

Sample size	Sample size was determined from similar experiments in the literature (ref1,2) and for all experiments a minimum of three technical replicates were analyzed per sample. For in vivo studies, the sample size was determined to be enough to obtain the statistical difference between groups. All sample sizes are listed in the corresponding figure legends or on the figures.<br>Reference:<br>1.Jiayu Jin., et al.BACH1 controls hepatic insulin signaling and glucose homeostasis in mice.Nat Commun. 2023; 14: 8428. doi: 10.1038/s41467-023-44088-z; PMCID: PMC10739811; PMID: 38129407<br>2.Qiao A., et al. Sam68 promotes hepatic gluconeogenesis via CRTC2. Nat Commun. 2021 Jun 7;12(1):3340. doi: 10.1038/s41467-021-23624-9. PMID: 34099657; PMCID: PMC8185084.
Data exclusions	No data were excluded from the analyses.
Replication	Data are presented as the mean ± SD. Replicates of experiments are specified in figure legends
Randomization	All samples and animals are randomly divided into different experimental groups as indicated.
Blinding	For mouse study, investigators were blinded to group allocation during data collection and analysis. For other studies, experiments were performed blinded.

Reporting for specific materials, systems and methods

We require information from authors about some types of materials, experimental systems and methods used in many studies. Here, indicate whether each material, system or method listed is relevant to your study. If you are not sure if a list item applies to your research, read the appropriate section before selecting a response.

Materials & experimental systems

n/a	Involved in the study
	☒ Antibodies
	☒ Eukaryotic cell lines
☒	☐ Palaeontology and archaeology
	☒ Animals and other organisms
☒	☐ Clinical data
☒	☐ Dual use research of concern
☒	☐ Plants

Methods

n/a	Involved in the study
☒	☐ ChIP-seq
☒	☐ Flow cytometry
☒	☐ MRI-based neuroimaging

Antibodies

Antibodies used

Primary antibodies used were RB (Cell Signaling Technology, # 9309, 1:1000), RB (Cell Signaling Technology, # 9313, 1:1000), E2F1 (Proteintech, #66515-1-Ig, 1:1000), Phospho-Rb (Ser795) (Cell Signaling Technology, # 9301S, 1:1000), RBL1 (Proteintech, #13354-1-AP, 1:1000), RBL2 (Proteintech, #27251-1-AP, 1:1000), Cyclin B1 (ABclonal, # A19037, 1: 1000), RNF40 (ABclonal, # A6443, 1: 1000), LRPPRC (ABclonal, # A3365, 1: 1000), CDK4 (ABclonal, # A11136, 1: 1000), CDK6 (ABclonal, # A0106, 1: 1000), KATNA1 (ABclonal, # A16491, 1: 1000), c-MYC (ABclonal, # A1309, 1: 1000), KLHDCS (Invitrogen, # PAS-54292, 1: 500), Vinculin (Santa Cruz, # sc-73614, 1:1000),β-Actin (Cell Signaling Technology, # 4970, 1:1000), Myc (Santa Cruz, # sc-40, 1:1000), HA (Cell Signaling Technology, # 3724, 1:1000), Flag (MBL, #M185-7, 1:1000), Rabbit IgG (ABclonal, # AS014, 1:3000), Mouse IgG (ABclonal, # AS003, 1:3000).

Validation

RB (Cell Signaling Technology, # 9309, 1:1000), https://www.cellsignal.cn/products/primary-antibodies/rb-4h1-mouse-mab/9309;
RB (Cell Signaling Technology, # 9313, 1:1000), https://www.cellsignal.cn/products/primary-antibodies/rb-d20-rabbit-mab/9313?site-search-type=Products&N=4294956287&Ntt=9313&fromPage=plp&_requestid=3172253l;
E2F1 (Proteintech, #66515-1-Ig, 1:1000), https://www.ptgcn.com/products/E2F1-Antibody-66515-1-Ig.htm#product-information;
Phospho-Rb (Ser795) (Cell Signaling Technology, # 9301S, 1:1000), https://www.cellsignal.com/products/primary-antibodies/phospho-rb-ser795-antibody/9301;
RBL1 (Proteintech, #13354-1-AP, 1:1000), https://www.ptglab.com/products/RBL1-Antibody-13354-1-AP.htm;
RBL2 (Proteintech, #27251-1-AP, 1:1000), https://www.ptglab.com/products/RBL2-Antibody-27251-1-AP.htm;
Cyclin B1 (ABclonal, # A19037, 1: 1000), https://abclonal.com.cn/catalog/A19037;
RNF40 (ABclonal, # A6443, 1: 1000), https://abclonal.com.cn/catalog/A6443;
LRPPRC (ABclonal, # A3365, 1: 1000), https://abclonal.com.cn/catalog/A3365;
CDK4 (ABclonal, # A11136, 1: 1000), https://abclonal.com.cn/catalog/A11136;
CDK6 (ABclonal, # A0106, 1: 1000), https://abclonal.com.cn/catalog/A0106
KATNA1 (ABclonal, # A16491, 1: 1000), https://abclonal.com.cn/catalog/A16491
Vinculin (Santa Cruz, # sc-73614, 1:1000), https://www.scbt.com/p/vinculin-antibody-7f9?requestFrom=search;
β-Actin (Cell Signaling Technology, # 4970, 1:1000), https://www.cellsignal.cn/products/primary-antibodies/b-actin-13e5-rabbit-mab/4970;
c-MYC (ABclonal, # A1309, 1: 1000), https://abclonal.com.cn/catalog/A1309;
KLHDC5 (Invitrogen, # PA5-54292, 1: 500), https://www.thermofisher.cn/cn/zh/antibody/product/KLHDC5-Antibody-Polyclonal/PA5-54292;
Myc (Santa Cruz, # sc-40, 1:1000), https://www.scbt.com/p/c-myc-antibody-9e10;
HA (Cell Signaling Technology, # 3724, 1:1000), https://www.cellsignal.cn/products/primary-antibodies/ha-tag-c29f4-rabbit-mab/3724;
Flag (MBL, # M185-7, 1:1000), http://www.mbl-chinawide.cn/uploads/pdf/M185-7-v2.pdf;
All of the antibodies used in this study were validated for the use in human specimens by the manufacturers and for the respective methods used in this manuscript (see home pages of respective manufacturers using catalogue numbers provided above).

Eukaryotic cell lines

Policy information about cell lines and Sex and Gender in Research

Cell line source(s)

293T, T24, UMUC3, UMUC14, Vcap, MDA-MB-231, MCF7, T47D, PC-3, C4-2, 22RV1 and 253J cells were purchased from ATCC (Manassas, VA).

Authentication

The cell lines were authenticated periodically via STR profiling (IDEXX BioResearch).

Mycoplasma contamination

All cell lines were tested negative of mycoplasma contamination.

Commonly misidentified lines
(See ICLAC register)

None commonly misidentified cell lines were used.

Animals and other research organisms

Policy information about studies involving animals; ARRIVE guidelines recommended for reporting animal research, and Sex and Gender in Research

Laboratory animals	6 weeks old NSG mice were used for bladder cancer xenograft study as described in the Methods section. Mice were housed in standard cages with an SPF environment with a 12-hour light/dark cycle at a room temperature of 22°C±2°C, humidity of 50%±5%, with free access to food and water.
Wild animals	No
Reporting on sex	The animals experiments were designed without any gender bias.
Field-collected samples	No
Ethics oversight	The animal study was approved by the Institutional Animal Care and Use Committee (IACUC) at the First Affiliated Hospital of Xi'an Jiaotong University (Xi'an, China).

Note that full information on the approval of the study protocol must also be provided in the manuscript.
