# Supplementary material for: MYC induces CDK4/6 inhibitors resistance by promoting pRB1 degradation
Source: Nat Commun. 2024 Feb 29;15:1871. doi: 10.1038/s41467-024-45796-w (PMC10904810; doi:10.1038/s41467-024-45796-w)
Supplement: Supplementary file 9 — Description of Additional Supplementary Files [file 41467_2024_45796_MOESM9_ESM.pdf]

Supplementary Data 1

1. Sequences of RT-qPCR primers
2. Sequences of ChIP-qPCR primers

Supplementary Data 2

1. The results for IgG, RB1 and MYC OE was listed in each sheet.
2. The gene ID was mapped in “IDmapping” sheet.
3. The analyzed results were listed in "Analyzed results"

Supplementary Data 3

1. The molecular glue screening library and molecular structure.

Supplementary Data 4

1. The killing ability analysis of drugs to HL60 cells

Supplementary Data 5

1. The ability of drugs to degrade c-Myc protein was detected
